# Deep learning approach to security enforcement in cloud workflow orchestration

**DOI:** 10.1186/s13677-022-00387-2

**Published:** 2023-01-18

**Authors:** Hadeel T. El-Kassabi, Mohamed Adel Serhani, Mohammad M. Masud, Khaled Shuaib, Khaled Khalil

**Affiliations:** 1 Department of Computer Science and Software Engineering, Gina Cody School of Engineering and Computer Science, Concordia University, Montreal, Canada; 2 College of Computing and Informatics, Sharjah University, Sharjah, UAE; 3 College of Information Technology, UAEU, Al Ain, Abu Dhabi UAE; 4 Faculty of Applied Science & Engineering, University of Toronto, Toronto, Ontario Canada

**Keywords:** Cloud, Cloud workflow, Security enforcement, Deep learning, Anomaly detection, Prediction, Covid-19

## Abstract

Supporting security and data privacy in cloud workflows has attracted significant research attention. For example, private patients’ data managed by a workflow deployed on the cloud need to be protected, and communication of such data across multiple stakeholders should also be secured. In general, security threats in cloud environments have been studied extensively. Such threats include data breaches, data loss, denial of service, service rejection, and malicious insiders generated from issues such as multi-tenancy, loss of control over data and trust. Supporting the security of a cloud workflow deployed and executed over a dynamic environment, across different platforms, involving different stakeholders, and dynamic data is a difficult task and is the sole responsibility of cloud providers. Therefore, in this paper, we propose an architecture and a formal model for security enforcement in cloud workflow orchestration. The proposed architecture emphasizes monitoring cloud resources, workflow tasks, and the data to detect and predict anomalies in cloud workflow orchestration using a multi-modal approach that combines deep learning, one class classification, and clustering. It also features an adaptation scheme to cope with anomalies and mitigate their effect on the workflow cloud performance. Our prediction model captures unsupervised static and dynamic features as well as reduces the data dimensionality, which leads to better characterization of various cloud workflow tasks, and thus provides better prediction of potential attacks. We conduct a set of experiments to evaluate the proposed anomaly detection, prediction, and adaptation schemes using a real COVID-19 dataset of patient health records. The results of the training and prediction experiments show high anomaly prediction accuracy in terms of precision, recall, and F1 scores. Other experimental results maintained a high execution performance of the cloud workflow after applying adaptation strategy to respond to some detected anomalies. The experiments demonstrate how the proposed architecture prevents unnecessary wastage of resources due to anomaly detection and prediction.

## Introduction

Cloud computing has emerged as a promising and powerful paradigm for managing and delivering computations, applications, and services over the Internet [1]. The processing power provided by the cloud covers a wide landscape of services, including storage, processing, and application services. This computational processing power has enabled researchers to use various computationally intensive and scientific workflows to perform vast experiments that were impossible to implement using local servers. This trend has significantly decreased the total cost incurred by related software systems [1] and is, in fact, a promising design paradigm for workflow deployment, processing, and orchestration. A typical large-scale scientific workflow comprises a set of interrelated tasks that are inherently complex, fault tolerant, and dynamically executed and orchestrated to produce scientific results. However, a cloud workflow refers to a workflow that is deployed and executed on the cloud. Cloud workflow features are classified as transparent, scalable, multi-tenant, and monitored in real-time [2].

With virtually infinite computing resources, cloud computing meets the needs of complex scientific data-intensive workflow tasks and releases cloud workflows from the burden of planning for resource provisioning. However, many research challenges need to be addressed before this potential can be fully realized. Such challenges include cloud security threats against integrity, authorization, availability, reliability, and trust. These challenges also apply to workflow security and privacy enforcement in the cloud environment, which is characterized by complexity, dynamicity, and multi-dimensional aspects. Supporting secure access, deployment, execution, and management of workflows over cloud platforms is of prime importance to both cloud providers and consumers. Providers must ensure that the resources they make available for workflow execution are not hacked, misused, or damaged. Similarly, workflow customers must be assured that their workflows and associated data are secured and protected from any outsider attacks.

Several cloud computing security threats have been identified [3] and extensively studied [4–8]. Such threats, which include data breaches, data leaks, data loss, denial of service (DoS), and malicious insiders, are generated from issues such as multi-tenancy, loss of control over data, and breaches of trust. Supporting security in a dynamic environment, across different platforms, different stakeholders, and various processes requires the involvement of various entities besides the cloud providers. A comprehensive security solution that considers security enforcement, trust chain in clouds, and ensures policies and regulations to guarantee security and privacy across multi-participants and heterogeneous environments is of paramount importance.

Emerging security issues in a cloud workflow have motivated researchers to address various research problems related to the security enforcement in a dynamically executed and orchestrated cloud workflow. However, most existing research on workflow management primarily tackled mainly aspects related to 1) anomaly and error detection [9], 2) workflow task scheduling [10], and 3) autonomic workflow resource provisioning and management [11]. In these studies, the main purpose was to avoid failure or resource contention and ensure efficient deployment, execution, and performance guarantee of these workflows [12]. Such initiatives neglect cloud workflow security enforcement which may strengthen the aspects mentioned above and fill the gap in handling security and data integrity of dynamic cloud workflows. Very few studies have exclusively tackled the security of cloud workflow orchestration, management, and enforcement [13]. Such studies typically focus on anomaly detection and prediction using various techniques such as HTM [14], statistical-clustering [8, 15, 16], regression [17, 18], and unsupervised machine learning (ML) [19]. They neither clearly define the security attributes nor specify the cloud workflow characteristics, which can be described as resource-aware, time-series, and highly dynamic. In addition, they focus on a specific security dimension, i.e., data, task, or resource of the workflow, and ignore other dimensions that will lead to better security enforcement when combined. Furthermore, the anomaly prediction and detection schemes proposed in most of these studies rely only on the dynamic features of the cloud workflow and neglect the static features that generally capture important workflow information and its hosting environment. Finally, most previous attempts primarily focused on security/anomaly detection and prediction and ignored the resources and workflow adaptation strategies that should be undertaken to mitigate security threats and possible attacks.

Therefore, to address some of the limitations of previous studies, we emphasize security anomaly/attack detection and prediction in a cloud workflow orchestration environment. We propose an adaptation scheme to cope with possible vulnerabilities and mitigate their effects on cloud workflow execution. In such an environment, the attack could target different entities and components such as workflow data, tasks, resources, monitoring, and adaptation. Our proposed model contributes to the state-of-art literature on cloud workflow security by including the following:

- A multidimensional security enforcement emphasizing cloud workflow security at various levels: the task, data, resources, and monitoring scheme.
- A scheme combining static and dynamic features for anomaly/attacks prediction, which is a unique way to model features that provide better anomaly detection, i.e., features that capture all stakeholders’ needs and all aspects of the cloud workflow. This scheme also applies deep learning autoencoder-based dimensionality reduction for the dynamic data, which will lead to better characterization of workflow tasks and will thus provide better attack prediction.
- An unsupervised learning technique that does not require class labeling, no additional work, and no manual intervention from experts, thus making it convenient and more realistic to deal with unknown anomalies.
- An adaptation model that accommodates the flexible representation and planning of resource requirements over time and over the various phases of the cloud workflow execution cycle.

The remainder of this paper is organized as follows: In Section 2 related work is discussed and compared and their limitations are identified. Section 3 presents a case study using a workflow based on a COVID-19 dataset. An architecture to enforce end-to-end security in a cloud workflow orchestration is proposed in Section 4. In section 5, a detailed cloud workflow security enforcement model formulation and the associated learning pipeline algorithms are presented. Section 6 presents the implementation details, conducted scenarios, experiments, and a discussion of the obtained results. Finally, conclusions and suggestions for future work are presented in Section 7.

## Related work

Security in cloud computing ecosystem is a comprehensive field that attracted significant attention as the cloud services hype grew exponentially, and cloud security threats and vulnerabilities also evolved over time. To help advance stat-of-the-art security solutions, first, related risks in emerging cloud services need to be identified. Such security risks are initiated by three types of attack vectors: external users, internal users, and cloud providers [20, 21]. Security threats in a cloud service environment are emerging over time. The most common threats include data breaches, data loss, DoS, malicious insiders, service traffic hijacking, shared technology vulnerabilities, malware, cyber-attacks, network intrusion, VM-level threats and data transparency [22–25].

Recently, deep learning approaches for cloud security threat detection have been proposed by several researchers. However, these approaches are unable to deliver a comprehensive solution for all security threats. However, they only address and detect patterns for a particular threat only in single deployment. The authors in [26] used a multi-layer neural network to detect and recognize malicious behaviors exhibited by users. They converted user behavior data into an understandable format and classified the malicious behavior for detection and recognition.

In [27], the authors proposed PredictDeep, a security analytical framework for known and unknown anomaly detection and prediction in Big Data systems. PredictDeep is proposed as a service to be offered to cloud users. The framework comprises three main modules, namely: a graph model designer, a feature extractor, and an anomaly predictor. PredictDeep is scalable and works well in a dynamic environment to monitor anomalies in real-time systems. However, with PredictDeep, anomaly detection assumes that all log files are accurate and no fake data that could compromise the accuracy of the prediction model has been injected. In addition, the proposed approach assumes the integrity of the infrastructure used for deployment.

Intrusion detection systems (IDS) are considered important tools for monitoring networks, services, and workflows for violations or malicious activities in cloud services orchestration. Detecting novel attacks in such scenarios is a challenging task. Deep learning-based intrusion detection techniques have yielded encouraging results relative to predicting unknown attacks and detection mechanisms. The authors in [28] presented an IDS using deep reinforcement learning-based architecture that can address and classify new and complex attacks. They employed a reward vector such that a classifier giving an identical result is awarded a positive point otherwise a negative point is obtained. In addition, the authors in [29] addressed a multi-cloud cooperative intrusion detection system and enhanced decision making in a real-time environment using a deep neural network (DNN) model. They employed historical feedback data to predict suspicious intrusions. Furthermore, a deep learning-based IDS has been proposed [25] to detect suspicious attacks in a cloud computing environment by monitoring network traffic. This system employs a self-adaptive genetic algorithm (SAGA) that automatically creates a DNN-based anomaly network IDS and demonstrated high detection rates, high accuracy, and low false alarm rates.

Other research initiatives have addressed cloud workflow security enforcement and several researchers and industries have proposed possible solutions to enhance the security of cloud services and cloud workflow orchestration. For Example, in [30], the developers currently working on the European-funded ASCLEPIOS (Advanced Secure Cloud Encrypted Platform for Internationally Orchestrated Solutions in Healthcare) project have exploited various cryptographic and access control techniques to protect user data privacy and provide protection against other security breaches as part of a cloud-based eHealth framework. One objective of this project is to enable various healthcare stakeholders to share data securely while preserving participant privacy. The proposed architecture consists of seven layers to provide security and analytic features to support data privacy and access control. However, the utilization of ML techniques for the detection, evaluation, and mitigation of potential anomalies in data or attacks on certain components of the overall system such as available resources was not incorporated into the proposed architecture.

In the PICASO project [31], a framework was proposed to enable cross-organization sharing of electronic health records using a cloud-based solution. This project aims to provide the required security and privacy measures in addition to service orchestration and data capture and management. Here, security features were implemented via separate subsystems to guarantee the privacy of patient records, user authentication, transaction information traceability, and enforcement of access control policies. However, the project did not implement any mechanisms for to detect of anomalies within the proposed framework.

Authors in [32] provided a literature review on security in FaaS orchestration systems [32]. They have classified the existing works considering various criteria including the protected asset, the cause of threats, and the protection approach. They concluded that most of the work focused on data confidentiality, however, data integrity is less considered. Function flows and platforms misconfiguration are also considered in most of the reviewed works. Moreover, authors in [33] provided another classification for existing works using Machine learning and Deep learning techniques for online malware detection in cloud. They classified the malware detection approaches into static analysis that is offline with no monitoring required, and dynamic analysis, where it requires real-time monitoring and use neural networks to predict when a virtual machine might be infected, this is more appropriate for cloud environment. The experimentation showed that Deep learning techniques provide good accuracy while detecting malware. However, this work did not analyze end-to-end security enforcement for workflows over cloud environment.

Zarca et al. proposed a semantic-aware and policy-driven security orchestration framework for autonomic security orchestration in IoT systems to detect semantic conflicts during the orchestration. The authors also proposed an optimized Service Function Chaining algorithm, which maximizes the QoS, security aspects and resources usage during Virtual network Security Functions allocation. However, they only detect anomalies but no prediction of anomalies is proposed [34].

Behavior attacks detection were proposed in [35] where different machine learning techniques for supervised classification were analyzed and compared. The study concluded that neural network models have the best performance in terms of accuracy to detect the impact malware on the process level features of virtual machines in the cloud. They collect different system features such as memory, CPU, and input/output from all process that are running on the VM at certain times. Similar work was proposed in [36] concentrating on different Convolutional Neural Networks (CNNs) for online detection of malware in cloud IaaS in real-time. The work focused on behavioral data using process level performance metrics including CPU, memory, and disk usage. Although their solution provide high accuracy, their proposed malware detection system is limited to a single virtual machine and does not support features such as auto-scaling [36].

Table 1 summarizes the state-of-the-art research with respect to anomaly detection in cloud environment. In this table we compare existing systems against the anomaly type they detect, anomaly detection technique adopted, data collected for anomaly detection, and other supported features such as real-time and orchestration. None of the surveyed systems provided anomaly detection from an end-to-end workflow orchestration perspective, rather a few systems detect anomalies in single VMs or single processes within a workflow [30, 34].

**Table 1:** Overview and classification of existing work on security in cloud

	Anomaly Types			Anomaly Detection Technique			System Data Collection	Real-time Anomaly Detection	Workflow Orchestration
Papers	Data Security	Behavior	Intrusion Detection	ML	Neural Networks	Policy-based			
**[**35**,** 36**]**		✓			✓		✓	✓	
**[**34**]**			✓			✓	✓	✓	✓
**[**37**,** 26**]**		✓			✓				
**[**28**]**			✓		✓				
**[**29**,** 25**]**			✓					✓	
**[**30**]**	✓			✓					✓
**[**31**]**	✓					✓		✓	
**Our System**	✓	✓	✓	✓	✓		✓	✓	✓

With the rapid increase in the use of cloud computing for electronic health records, issues related to security and privacy are critical for establishing participant trust in the deployed system. Thus, complex data-intensive cloud workflows must provide trustworthy results that enforce secure input and output data free of unauthorized access and malicious manipulation. Challenges related to handling security breaches in a cloud workflow orchestration system include the ability to identify the properties of each specific task comprising the workflow and each physical resource allocated in this dynamic infrastructure as well as integrate the collected information to detect anomalies and malicious actions. Anomaly detection must be timely, and an appropriate remedial action must be selected and executed before damage occurs. Moreover, the security process must have a minimal effect on the computing environment to maintain workflow execution performance. In other words, we must guarantee smooth and efficient handling of all security breaches including identification, prediction, and remediation.

To support security enforcement of cloud workflows and address some of the above abovementioned research challenges, we approach this problem from different dimensions including multi-level security enforcement, pre-evaluation of various prediction models for security threat detection and prediction, combining static and dynamic features for anomaly detection, and adaptation strategies to mitigate various security risks. Before we detail the features of our proposed cloud workflow security enforcement methodology, we illustrate the concept with an example cloud workflow handling COVID-19 dataset and identify potential security threats the cloud workflow may encounter that our proposed approach is expected to help detect. In addition, we predict some of these breaches and propose adaptation actions to protect against them.

## Case study: COVID-19 cloud workflow

The effectiveness of healthcare systems worldwide has been challenged recently owing to the outbreak of the Novel Coronavirus (COVID-19) which was declared a pandemic by the WHO in March 2020. The impact of such new strains of viruses has been demonstrated to defeat all expectations of any healthcare system. This pandemic has strained involved entities, working to find a cure or vaccine, including healthcare providers, government agencies, and research facilities. Such pressures have led to proper protection of facilities, confidential data, and workflows from possible vicious attacks that could easily compromise the integrity of the overall process [38]. During COVID-19 pandemic, the reliance on online resources and cloud-based infrastructure systems has increased drastically due to lockdowns, contact-tracing applications, and increased use of remote working and distance-learning platforms. This has caused a huge leap in cyber-attacks and data confidentiality and integrity breaches [38]. To illustrate the applicability and usefulness of the security enforcement architecture and identify the main security threats in cloud workflow orchestration, we describe a case study involving a cloud workflow encompassing the composition of tasks handling a COVID-19 dataset.

### Cloud workflow and COVID-19 dataset

**Fig. 1:**
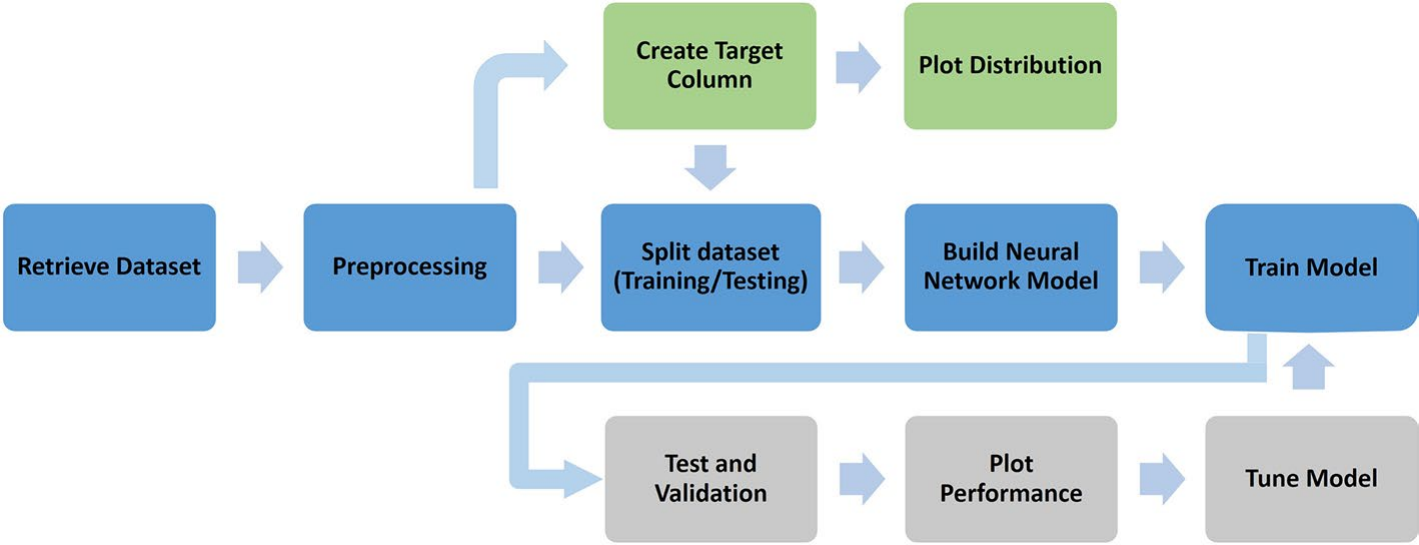
COVID-19 patient health monitoring workflow example

Figure 1 shows the health monitoring cloud workflow we developed using the epidemiological data from a COVID-19 outbreak dataset that employs a deep learning model to predict the length of hospital stay of COVID-19 patients [39]. The dataset was collected and curated from national, provincial, and municipal health reports, as well as other online reports. The data are geocoded and include symptoms, key dates (date of onset, admission, and confirmation), and travel histories of different patients [40]. We used data collected up to the June 20, 2020, including 2,500,000 records, each of which represents an individual patient case. The dataset includes 33 columns including patient ID, age, gender, date_onset_symptoms, date_admission_hospital, date_confirmation, additional_information, chronic_disease_binary, chronic_disease, symptoms, and outcome. The explanation for each field is provided in [39]. We adopted this cloud workflow example to identify and evaluate different security breaches that could be encountered and therefore mitigate their effects. The workflow was deployed on a Docker Swarm Cluster and the data were loaded automatically from database tables to satisfy the service tasks outlined in the workflow. The workflow comprises a set of both sequential and parallel tasks. The sequential tasks include retrieving data from the database and conducting data processing, while the parallel tasks include training, prediction, and validation tasks.

### Security threats

Different security issues in a cloud-based infrastructure were addressed in the literature, examples of breaches include for instance insider attacks, data loss, and DoS attacks. In this section, we focus on anomaly detection in a cloud workflow orchestration setting. In such an environment, an attack could target different entities and components including workflow data, tasks, resources, monitoring, and adaptation components. In what follows, we describe a few examples of security breaches in a cloud workflow.

#### Cloud workflow data attack

Some examples of data attacks involve data injection attacks that intend to corrupt the dataset or compromise it through, for example, suspicious sharing or downloads. Other anomalies include unauthorized data access and anomalous admin user activities. For example, in our cloud workflow, the attacker may inject redundant and fabricated data into the workflow to tamper with training and prediction processes which will affect the quality of the prediction model and may cause critical problems (e.g. patient death) or overburden the ML training process, thereby falsely activating Quality of Service (QoS) degradation and triggering unnecessary workflow adaptation.

#### Cloud workflow task attack

Cloud workflow is composed of many different tasks that can run in parallel or sequentially with different dependency levels. Workflow task attacks include a wide range of different anomalies including malware infection, query injection, and DoS. Furthermore, an attacker can maximize damage, by targeting sensitive processes or tasks (e.g., tasks on which many other tasks depend).

#### Resource attack

Resources such as cloud VMs, CPUs, memory, and networks can also be the target of different types of attacks, including unauthorized resource access, or overwhelming service requests. Such attacks could arise by falsely reporting resource overload/overutilization in monitoring logs, which will cause the compromised node to trigger unnecessary and costly workflow adaptation processes.

#### Monitoring and adaptation component attack

Monitoring and adaptation component attacks are very crucial in any cloud workflow orchestration environment because these components are crucial to resource management and performance optimization. In this workflow example, an attack against a monitoring system can force the compromised monitoring task to generate false resource underutilization logs, to avoid necessary adaptation and thus, causing performance degradation leading to a DoS. Another example of such an attack is automatic system reconfiguration which can cause a compromised node to falsely identify a problem and trigger unnecessary adaptation actions.

The aforementioned attack types negatively impact the performance and integrity of a cloud workflow orchestration system. In this work, we focus on anomaly detection in cloud workflow data, cloud resources, tasks, and monitoring components. Hence, we propose to monitor resources, such as utilization of CPU, memory, I/O, and network, as well as task profile, and task performance. In the following section, we present our proposed security enforcement for cloud workflow orchestration.

## End-to-end security enforcement in cloud workflow orchestration

**Fig. 2:**
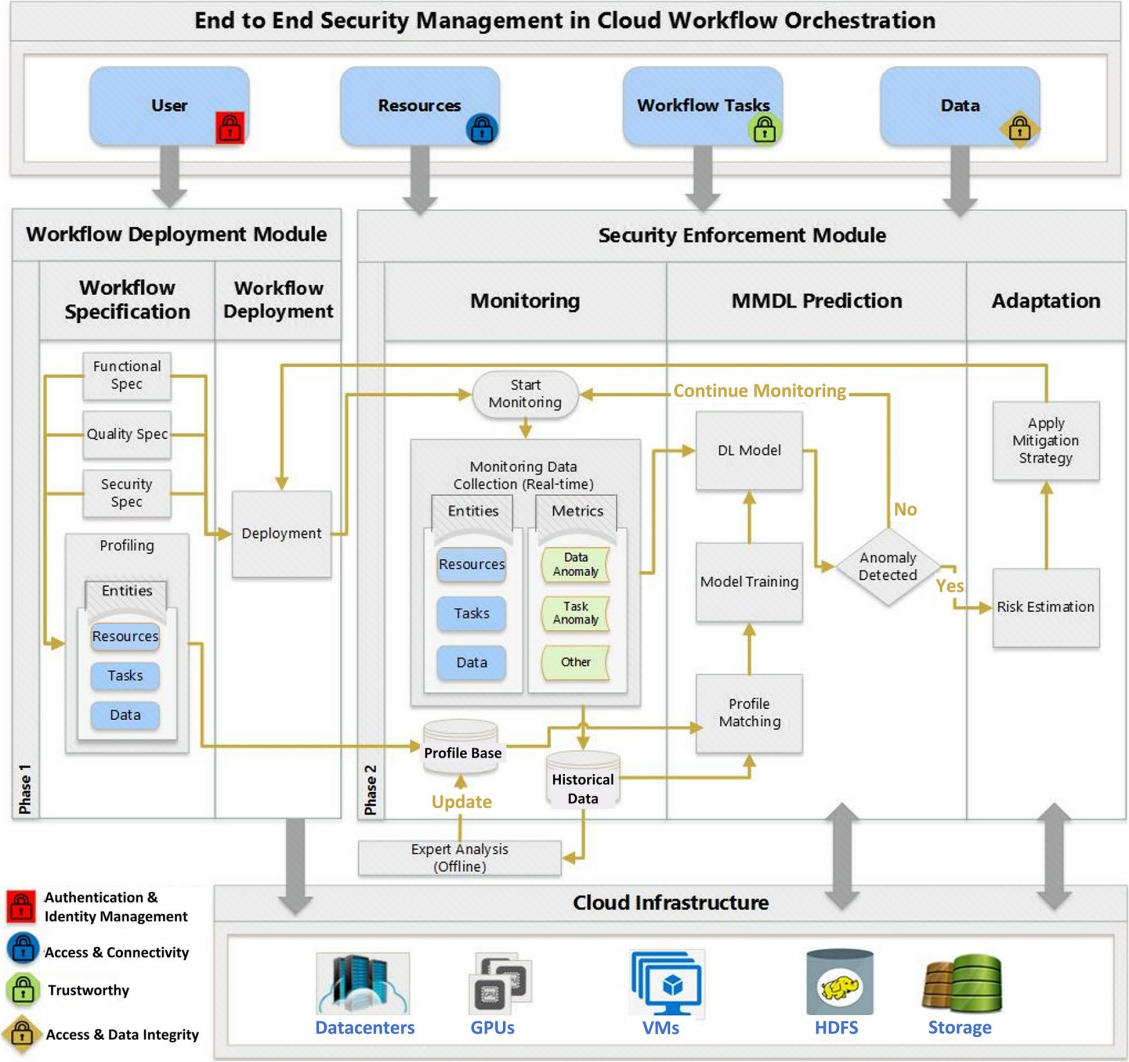
An architecture for security enforcement in cloud workflow orchestration

In this section, we design and describe our end-to-end security enforcement architecture as depicted in Fig. 2. It consists of two main modules: a workflow deployment module and a security enforcement module. Both modules use the underlying processing and storage resources (e.g., VMs, GPUs, Storage) from the cloud infrastructure to dispatch various storage and processing tasks. Security enforcement events implemented within our architecture are applied to four main entities: the user, resources, workflow tasks, and data.

In the following, we describe each component of the architecture in detail and highlight the security features that enhance security, data integrity, and authentication.

### Entities

Entities interact with the two modules of the architecture to ensure various security boundaries including authentication and identity management for users interacting with the architecture, access and connectivity management of the employed resources, security enforcement of cloud workflow tasks, and workflow data access and integrity.

### Workflow deployment module

This module involves two sub-components, the workflow specification, and the workflow deployment components. The workflow specification component builds the functional and non-functional (quality and security) requirements of the workflow and creates profiles for entities, such as tasks, data, and resources. The workflow deployment component manages the workflow deployment and execution lifecycle over the cloud infrastructure. The output of this module is a running workflow monitored by the security enhancement module to detect and/or predict encountered security threats and adopt the necessary adaptation action to mitigate it.

### Security enforcement module

This module is composed of three sub-components: monitoring, Multi-Modal Deep Learning Autoencoder (MMDLA) based prediction and adaptation sub-modules. These sub-modules mutually interact to achieve a complete scenario of cloud workflow monitoring, anomaly detection, and prediction. Finally, these submodules apply an adaptation strategy to mitigate risks identified through various anomaly evaluations.

### Monitoring sub-module

This submodule is responsible for continuous data collection and monitoring. Various runtime data/logs are collected from monitored entities including tasks, data, and resources. The collected data are used for the training, and prediction purposes and are stored in a historical database for further analysis.

### MMDLA sub-module

This module uses the data collected from the monitoring submodule as an input to train a multi-modal deep learning autoencoder model for dimension reduction and trains a profile matching classification model using the dimensionally reduced data to predict anomalies. Training of the MMDLA model employs a combination of the input data generated from the entities profiling module (static) and monitoring time-series real-time logs data (dynamic). The resultant MMDLA model reduces the data dimension to increase efficiency and efficacy and provides reduced dimensional data as input to an anomaly detection ML algorithm to accomplish anomaly detection. If an anomaly is detected, then anomaly evaluation is performed to determine the type and threat level of the detected anomaly. Then, the anomaly evaluation information is passed as input to the risk estimation process and eventually stored in a database for expert validation (e.g., to identify suspicious user behavior). A detailed description and implementation of the key component’s features of this module is given in subsequent sections.

### Adaptation sub-module

This submodule implements adaptation strategies to proactively react to security threats before they occur and propagate. This begins by estimating the risk of each anomaly detected/predicted by the previous module to ultimately apply a mitigation strategy which may involve a redeployment of the cloud workflow to handle the employed adaptations. Such an adaptation may involve securing access to cloud workflow resource execution, guaranteeing legitimate additional resource allocation or deallocation, and terminating compromised tasks.

### Cloud infrastructure

This serves the architecture requirements in terms of the various resources needed to process and store data. Processing tasks include MMDLA model training for dimension reduction, anomaly detection model training and classification, and data storage monitoring.

## Cloud workflow security enforcement module

In this section, we detail the working principle of the MMDLA prediction-based security enforcement module. First, we define essential terms in understanding the prediction model, and then we discuss problem formulation. Finally, we describe the learning pipelines algorithms used for the solution approach.

### Definitions

**Definition 1 (Task)** *A task* $\mathcal{T}$ *is an operational unit consisting of one or more instructions, and can be dependent on one or more other tasks. Each task* ${\mathcal{T}}_i$ *runs on a designated container* ${\mathcal{C}}_i$ *such as a virtual machine.*

**Definition 2( Workflow)** *A workflow* $\mathcal{W}$ *is a collection of tasks {* ${\mathcal{T}}_1$*, ...,* ${\mathcal{T}}_n$ *} performed according to a schedule* $\mathcal{S}$ *toward achieving a specific work (*e.g.*, patient classification).*

**Definition 3 (Task Profile)** *A Task profile* ${\mathcal{P}}_i$ *of a task* ${\mathcal{T}}_i$ *is the tuple (δ*_*i*_*,* ${\mathcal{R}}_i$*) where δ*_*i*_ *is the unique id of the task and* ${\mathcal{R}}_i$ *is the task runtime data, to be defined next.*

**Definition 4 (Task runtime data)** *Task runtime data* ${\mathcal{R}}_i$ *of task* ${\mathcal{T}}_i$ *consists of both static and dynamic runtime data, which can be represented as a tuple (D, λ, η, μ, Θ)* _*i*_*. The static runtime data are composed of first four items of the tuple, namely:*

*D*_*i*_: The task duration in seconds.

*λ*_*i*_: The task category (e.g. preprocessing, training, evaluation).

*η*_*i*_: The input size in bytes.

*μ*_*i*_: The output size in bytes.

The dynamic runtime data Θ_*i*_ is a multivariate time-series data produced by a task monitoring system for task ${\mathcal{T}}_i$ which consists of periodical observation of six different runtime parameters, namely, CPU utilization, memory consumption, network input, network output, disk read, and disk write. Therefore, Θ_*i*_ can be defined as the tuple (Π, $\mathcal{M}$, $\mathcal{A}$, $\mathcal{B}$, $\mathcal{D}$, $\mathcal{E}$) _*i*_, as explained below.

Each observation of dynamic runtime data is performed every *τ* seconds (a system parameter). Therefore, the total number of such observations for ${\mathcal{T}}_i$ is

1 $${k}_i=\frac{D_i}{\tau }$$

where *D*_*i*_ is the task duration as mentioned above. The six time-series variables are as follows:

1. Π_***i***_: The CPU utilization observations, which generate a time-series data such that, Π_***i***_={***π***_***i***_[1]**,** ... ***π***_***i***_[***k***_***i***_]}, where ***π***_***i***_[***j***] is the ***j***-th observation of CPU utilization for task 𝒯𝑖.
2. ℳ_𝑖_: The series of memory usage observations performed every ***τ*** seconds, ***M***_***i***_ = { ***m***_***i***_[1],..., ***m***_***i***_[***k***_***i***_]}**.**
3. 𝒜_𝑖_: The series of (cumulative) network input volume (in KB) observations for Container $\mathcal{C}_{\boldsymbol{i}}$ performed every ***τ*** seconds, $\mathcal{A}_{\boldsymbol{i}}=\left\{{\boldsymbol{\alpha}}_{\boldsymbol{i}}\left[\textbf{1}\right],\dots, {\boldsymbol{\alpha}}_{\boldsymbol{i}}\left[{\boldsymbol{k}}_{\boldsymbol{i}}\right]\right\}$  
4. $\mathcal{B}_{\boldsymbol{i}}$: The series of (cumulative) network output volume (in KB) observations for Container $\mathcal{C}_{\boldsymbol{i}}$ performed every ***τ*** seconds, $\mathcal{B}_{\boldsymbol{i}}=\left\{{\boldsymbol{\beta}}_{\boldsymbol{i}}\left[\textbf{1}\right],\dots, {\boldsymbol{\beta}}_{\boldsymbol{i}}\left[{\boldsymbol{k}}_{\boldsymbol{i}}\right]\right\}$
5. $\mathcal{D}_{\boldsymbol{i}}$: The series of (cumulative) disk read volume (in KB) observations for Container $\mathcal{C}_{\boldsymbol{i}}$ performed every ***τ*** seconds, $\mathcal{D}_{\boldsymbol{i}}=\left\{{\boldsymbol{d}}_{\boldsymbol{i}}\left[\textbf{1}\right],\dots, {\boldsymbol{d}}_{\boldsymbol{i}}\left[{\boldsymbol{k}}_{\boldsymbol{i}}\right]\right\}$
6. $\mathcal{E}_{\boldsymbol{i}}$: The series of (cumulative) disk write volume (in KB) observations for Container $\mathcal{C}_{\boldsymbol{i}}$ performed every ***τ*** seconds, $\mathcal{E}_{\boldsymbol{i}}=\left\{{\boldsymbol{e}}_{\boldsymbol{i}}\left[\textbf{1}\right],\dots, {\boldsymbol{e}}_{\boldsymbol{i}}\left[{\boldsymbol{k}}_{\boldsymbol{i}}\right]\right\}$

Therefore, dynamic runtime data Θ_*i*_ can be expressed as the following two-dimensional matrix:

2 $${\Theta}_i=\left\{{\Pi}_i,{\mathcal{M}}_i,{\mathcal{A}}_i,{\mathcal{B}}_i,{\mathcal{D}}_i,{\mathcal{E}}_i\right\}=\left(\begin{array}{llllll}{\pi}_i\left[1\right]& {m}_i\left[1\right]& {\alpha}_i\left[1\right]& {\beta}_i\left[1\right]& {d}_i\left[1\right]& {e}_i\left[1\right]\\ {}{\pi}_i\left[2\right]& {m}_i\left[1\right]& {\alpha}_i\left[2\right]& {\beta}_i\left[2\right]& {d}_i\left[2\right]& {e}_i\left[2\right]\\ {}\dots & \dots & \dots & \dots & \dots & \dots \\ {}{\pi}_i\left[{k}_i\right]& {m}_i\left[1\right]& {\alpha}_i\left[{k}_i\right]& {\beta}_i\left[{k}_i\right]& {d}_i\left[{k}_i\right]& {e}_i\left[{k}_i\right]\\ {}& & & & & \end{array}\right)$$

### Problem formulation

Let $R=\left\{{\mathcal{R}}_1,\dots {\mathcal{R}}_i,\dots, {\mathcal{R}}_n\right\}$ be the set of all task runtime information under a normal scenario, i.e., all runtime scenarios without any attacks. We assume that any attack would cause at least one running task ${\mathcal{T}}^{\prime }$ to behave in a manner that would generate the corresponding task runtime information ${\mathcal{R}}^{\prime }$ such that

3 $${\mathcal{R}}^{\prime}\notin R$$

Therefore, relation 3 is a necessary and sufficient condition for ${\mathcal{T}}^{\prime }$ being affected by an attack. So, the problem is to learn a model $\mathcal{H}\left(\mathcal{R}\right)$ that will predict whether any given task runtime information $\mathcal{R}$ has been generated by a task affected by an attack. Formally, the model $\mathcal{H}$, given input $\mathcal{R}$, outputs true or false such that:

4 $$\mathcal{H}\left(\mathcal{R}\right)=\left\{\begin{array}{l} true, if\ \mathcal{R}\notin R\\ {} false, otherwise\end{array}\right.$$

In other words, $\mathcal{H}\left(\mathcal{R}\right)$ will hold true if and only if $\mathcal{R}$ belongs to a task affected by an attack.

### Solution approach

To learn model $\mathcal{H}$ according to condition (4) above, we must train $\mathcal{H}$ with the generalized description of *R*, i.e., the set of all possible task runtime information generated by tasks not affected by any attack. Here, we employ an unsupervised technique for training, where we attempt to learn $\mathcal{H}$ from a subset of *R*, i.e., the set of all normal task runtime information. We collect the normal data from workflows running under normal scenarios, i.e., scenarios known to have no attacks. This data is then used to learn the desired model using one-class classifier learning techniques, including one-class SVM, isolation forest, elliptic envelope, and local outlier factor. We also use different clustering algorithms to learn clusters or normal data.

### Learning pipeline algorithms and descriptions

The learning process requires several steps in the learning pipeline, namely, monitoring data collection from logs, feature extraction and feature vector generation, feature dimension reduction, training, and classification. The following subsections describe these processes in detail.

#### Monitoring data collection

For each workflow, logs are generated by the task monitor of each container ${\mathcal{C}}_i$ of task ${\mathcal{T}}_i$; these logs are collected and processed for training. The logs are primarily represented in an unstructured text format, which must be processed and converted into a structured format.

#### Feature extraction and feature vector generation

The processed logs are then used to extract the task profile, which includes the task *id*, static runtime data and dynamic runtime data, as explained above. The extracted task profiles are then used to generate two types of feature vectors for each task.

The static feature vector *S*_*i*_ = (*D*, *λ*, *η*, *μ*) _*i*_ consists of the static runtime data of task ${\mathcal{T}}_i$, and the dynamic feature vector, (i.e., the feature matrix) is essentially the dynamic runtime data of ${\mathcal{T}}_i$, i.e., Θ_*i*_. Therefore, the combined feature vector for task ${\mathcal{T}}_i$ is essentially the task runtime data *R*_*i*_, as defined previously.

A training dataset *X*_*train*_ is built by collecting task runtime information from *n* tasks. In other words, ${X}_{train}={\cup}_{j=1}^n\left\{{\mathcal{T}}_j\right\}$. Therefore, the feature extraction process generates the training feature vector *R*_*train*_, consisting of the feature vectors of all tasks in *X*_*train*_, i.e.,

5 $${R}_{train}={\left\{{R}_1,\dots, {R}_n\right\}}^T$$

Where *R*_*i*_ is the feature vector of ${\mathcal{T}}_i$. Recall that *R*_*i*_ consists of two types of feature vectors, namely static feature vector (one-dimensional) *S*_*i*_ which is duplicated to be concatenated with each row in the dynamic feature vector (2D matrix) Θ_*i*_. Thus, we can represent *R*_*train*_ as a concatenation of two matrices:

6 $${R}_{train}=\left({S}_{train}\right)\left({\Theta}_{train}\right)={\left\{{S}_1,\dots, {S}_n\right\}}^T{\left\{{\Theta}_1,\dots, {\Theta}_n\right\}}^T=\left(\begin{array}{ll}{S}_1& {\Theta}_1\\ {}\dots & \dots \\ {}{S}_n& {\Theta}_n\\ {}& \end{array}\right)$$

#### Feature reduction using deep autoencoder

As discussed previously, the feature vector for each task consists of four static features and six time-series features. To train a model that learns from two-dimensional feature vectors, we need to flatten the time-series feature matrix to a one-dimensional feature vector and combine it with the static features. However, this can cause the formation of a very high-dimensional feature vector. In particular, the total features in the feature vector would be 4 + 6 *k*_*i*_, where *k*_*i*_ is the number of observations of the dynamic features. For example, if *k*_*i*_ = 100, the total number of flattened features would be 604. Therefore, we must adopt a feature reduction technique. Here, we reduce the number of features using an unsupervised deep learning technique called AutoEncoder [41]. Although there are many alternative feature reduction or feature selection techniques, we employ the AutoEncoder technique for two main reasons:

- First, AutoEncoder can perform unsupervised feature reduction, which is an important aspect of our proposed model.
- Second, we propose multi-modal deep learning (MMDLA) based AutoEncoder model by combining long short term memory (LSTM) (a specific type of recurrent neural network (RNN)) [42] with a Deep Feed Forward network (DFN). This MMDLA model facilitates the feature reduction process to learn from the temporal relationships among time-series features and combine it with static features, rather than implementing a feature reduction process that flattens all the time-series features and loses the temporal information contained in the feature set.

**Fig. 3:**
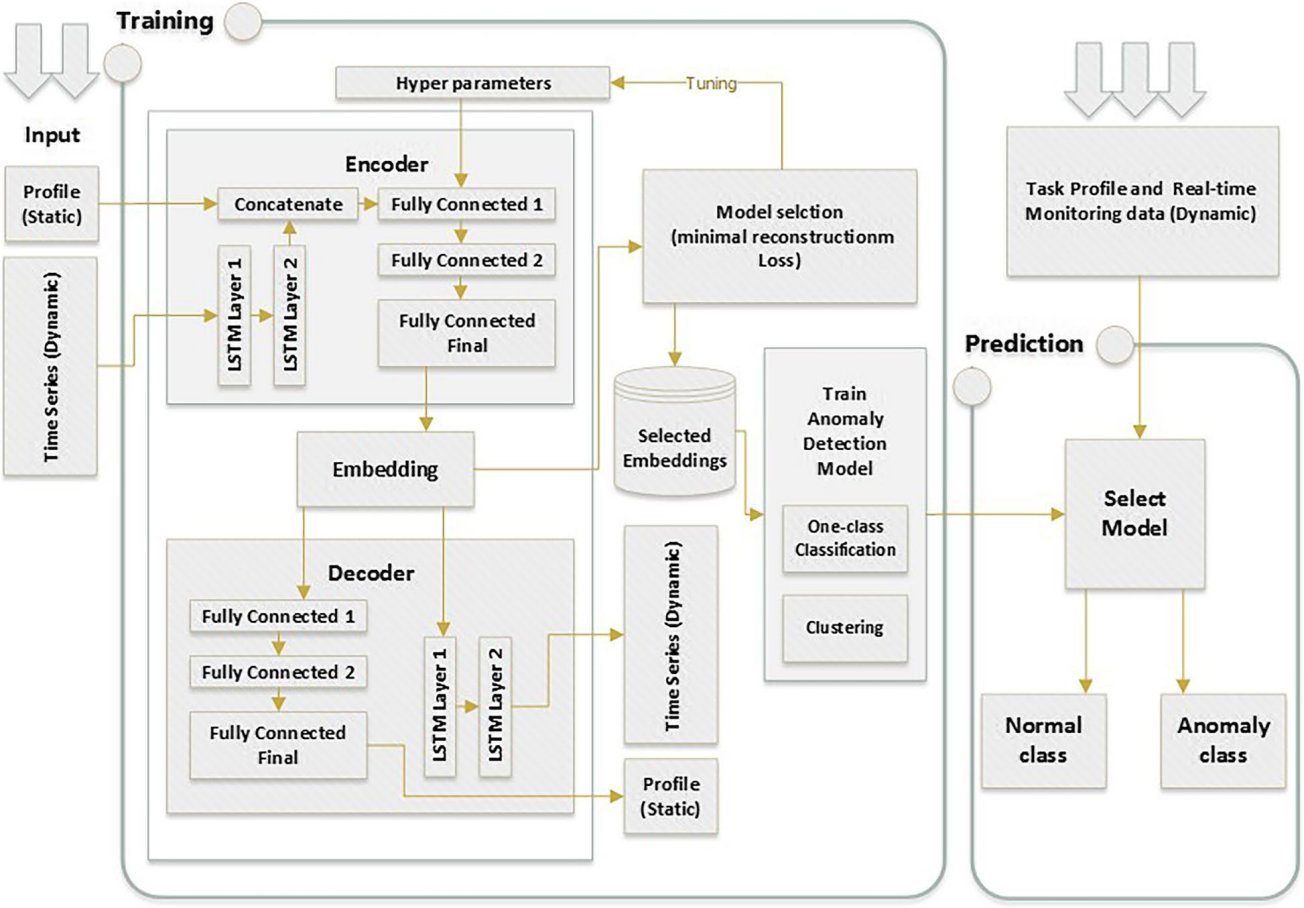
High level diagram of the proposed approach

Figure 3 shows the high-level architecture of this feature reduction, training, and prediction technique.

Here, we describe the proposed AutoEncoder-based model which will be referred to as MMDLA. It consists of two main components, namely, the Encoder, and the Decoder in detail. The Encoder consists of two LSTM layers, a concatenation layer, and three fully connected layers as shown in Fig. 3. The purpose of the Encoder is to take feature vector *R*_*train*_ as input, then output a reduced dimensional feature vector (also known as embedding) *R*_*ϵ*_.

The Decoder has a network concept similar to that of the Encoder. The purpose of the Decoder is to take the embedding *R*_*ϵ*_ as input and reconstruct the original feature vector. Thus, the output of the decoder is *R*′_*train*_ = (*S*′_*train*_)(Θ′_*train*_), such that the matrix dimensions of (*S*′_*train*_) and (Θ′_*train*_) are the same as those of (*S*_*train*_) and (Θ_*train*_), respectively. Essentially, (*S*′_*train*_) and (Θ′_*train*_) are approximations of (*S*_*train*_) and (Θ_*train*_), respectively. Therefore, the learning objective of the AutoEncoder is to minimize the loss, i.e., the difference between the input and reconstructed output. Therefore, the AutoEncoder loss $\mathcal{L}$ can be represented as the sum of the loss of the static runtime data (${\mathcal{L}}_{stat}$) and dynamic runtime data (${\mathcal{L}}_{dyn}$):

7 $$\mathcal{L}={\mathcal{L}}_{stat}+{\mathcal{L}}_{dyn}={\Sigma}_{i=1}^n{\left({S}_i-S{\prime}_i\right)}^2+{\Sigma}_{i=1}^n{\left({\Theta}_i-\Theta {\prime}_i\right)}^2$$

After training the AutoEncoder model, we take the reduced dimensional feature vector, i.e., embedding *R*_*ϵ*_ as the new feature vector and train an unsupervised anomaly detection model (e.g., one class classifier).

#### Classification and prediction

The embedded feature vector *R*_*ϵ*_ is used to train an anomaly detection model $\mathcal{H}$ as expressed in equation 4. The learning algorithm is assumed to be one-class classifier training or unsupervised clustering that only requires normal data for training. Once the clustering or one-class classifier model is trained, it is deployed in the system to detect (i.e., predict) anomalous task runtime data, supposedly generated from tasks affected by an attack.

### Algorithms

In this section, we present the algorithms for the learning and prediction processes of the security enforcement model. Algorithm 1 describes the training pipeline of the anomaly detection model. The input to this algorithm is the training data. First, lines 1–4 retrieve monitoring log data, extract features, and generate the feature vector. Then we train the AutoEncoder (lines 6–7). In line 8, we obtain the embedding of the training data from the AutoEncoder, and in line 9, an anomaly detection model is trained with this reduced feature vector.

**Figure:**
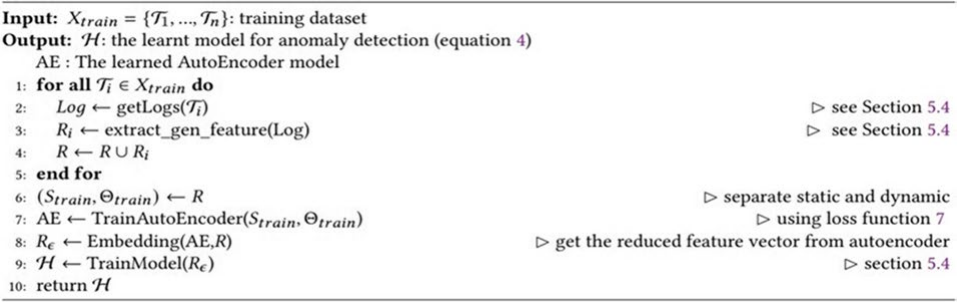
**Algorithm 1 **Security Enforcement Model Training (X_*train*_)

Algorithm 2 requires three inputs, namely, the task to examine, prediction model $\mathcal{H}$, and the AutoEncoder model *AE*. First, we extract features and generate a feature vector from the logs. Line 3 applies the embedding on the task runtime data to obtain a reduced feature vector. Finally, anomaly detection model $\mathcal{H}$ predicts whether the runtime data is generated by a task affected by an attack.

**Figure:**
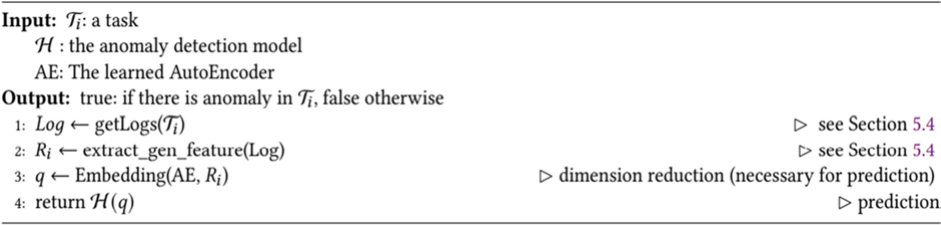
**Algorithm 2** Attack prediction (𝒯_𝑖_,𝓗,AE)

### Adaptation scheme

The monitoring process is performed continuously for all running cloud workflows. The monitoring logs are collected periodically, and the status of all cloud workflows is checked for anomalies or other quality performance issues such as performance degradation and resource over/under utilization. For each running cloud workflow, we inspect its tasks monitoring logs by running the attack prediction algorithm depicted in Section 5.5. Once an attack is detected, we apply the appropriate mitigation strategy including task restart, workflow restart, and reverting to former logs depending on the outcome of the risk estimation process. The risk estimation process evaluates the status of the workflow and cause of the anomaly and recommends mitigation action to prevent or override the attack. First, it checks the anomaly type and the task status, then recommends a set of actions according to the following rules. If the anomaly type is resource over-utilization, additional resources is allocated to the workflow. Otherwise, if the anomaly type is under-utilization, then a resource can be released. Different anomaly types are handled by the risk estimation process according to the predefined rules. The set of recommended actions can also be applied to the tasks in the task dependency list. For example, if a task was attacked, the task dependencies list is checked to decide whether other dependent tasks should be also restarted along with the task under attack. Otherwise, if no attack is detected, the performance recorded values of attributes are checked and if they do not satisfy the required quality thresholds, adaptation actions are applied (e.g., adding a new node if CPUs are over-utilized). Algorithm 3 presents the cloud workflow adaptation after an anomaly is detected. This algorithm takes as input the list of currently running cloud workflows, the anomaly detection model, the trained AutoEncoder, the collected monitoring logs, the desired/acceptable ranges for each performance quality feature, and the list of possible adaptation actions that will maintain the required workflow QoS levels. First, the algorithm applies an anomaly prediction model to each task in the workflow. When an anomaly is detected, a mitigation strategy is applied as explained in Section 4. Otherwise, if monitoring logs show out-of-range values, the regular adaptation mechanisms are applied.

**Figure:**
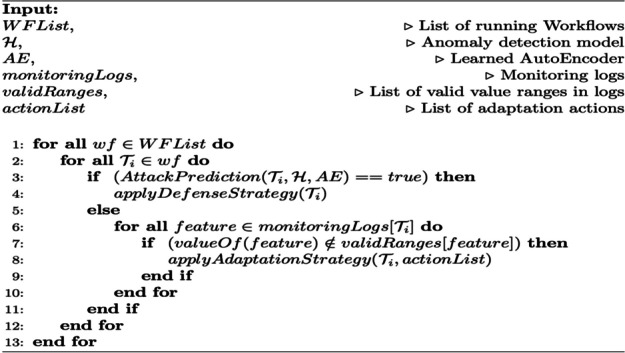
**Algorithm 3** Cloud Workflow adaptation with anomaly detection

## Implementation and experiment

### Environment setup

**Fig. 4:**
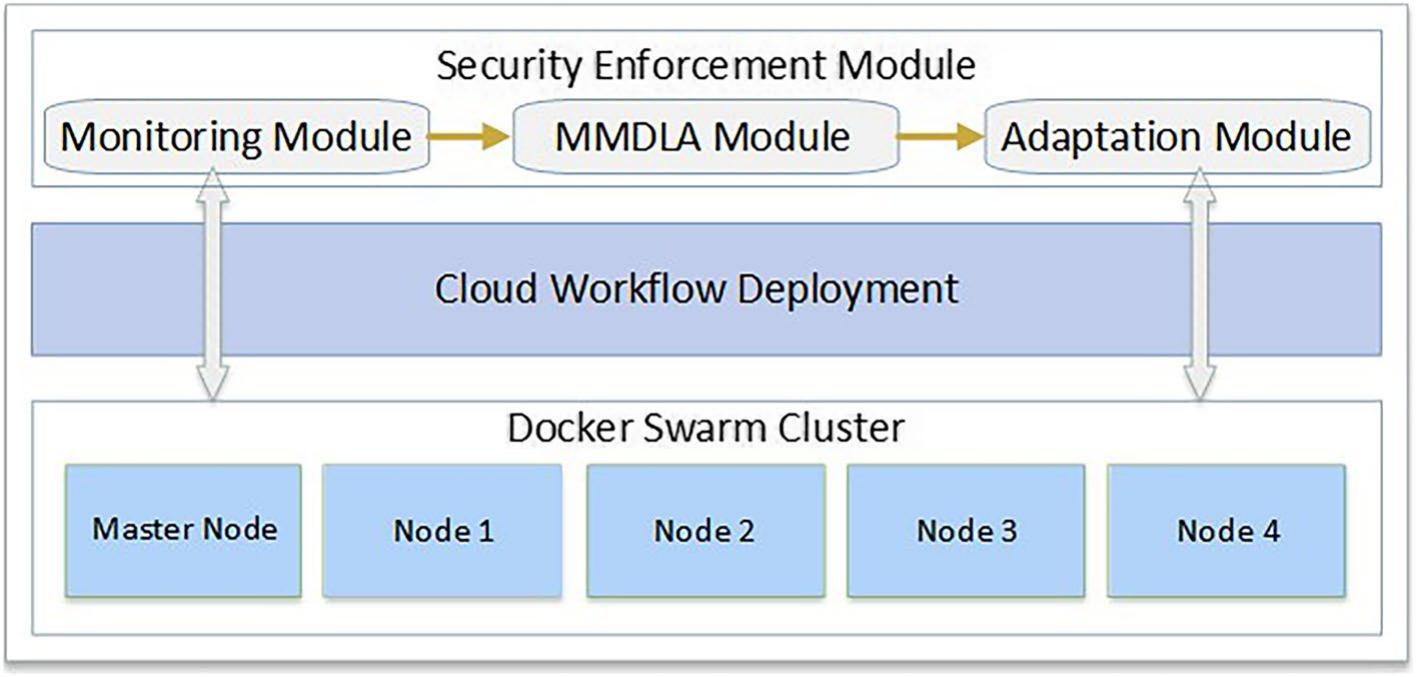
Experimentation environment

In this section, we describe the experimental environment. We created a Docker Swarm Cluster comprising one master node and four worker nodes. We deployed the cloud workflow described in Section 3 over a workstation running Linux Ubuntu 18.04 with 24 CPU cores and two NVIDIA GeForce GTX 1080 Ti GPUs with 11 GB GDDR5X memory each, a 1-TB HDD, and 64-GB RAM. Each task in the cloud workflow was created as a Docker container executed using different data input sizes. The Docker swarm cluster had a master node, that performed the orchestration to conserve the required cluster state. The worker nodes received and ran tasks dispatched from the master node. Deploying a workflow to a swarm requires providing service definition to the master node, which accordingly dispatches units of work, called tasks, to the worker nodes. During workflow execution, we collected a live data stream to run task containers to monitor various performance metrics, which are discussed in detail in the following section. Additionally, we ran other mock containers to overload nodes in the cluster to simulate a real environment. The experimental environment is depicted in Fig. 4.

We implemented the proposed algorithms in Jupyter Notebook running Python 3.6. The AutoEncoder and SVM algorithms were developed using Pytorch and Scikit-learn, which are open-source Python implementations of machine learning and deep learning neural networks. The experiments were executed on a Mac computer with OS X Catalina 10.15.4 operating system with a 2.8-GHz Quad-Core Intel Core i7 and 16-GB 1600-MHz DDR3 RAM.

### Dataset

#### Dataset description

In the experiments, we combined two types of data for each running task. We initially defined a static task profile to include various types of information such as task duration, data input size, data output size, and task category (pre-processing, training, or evaluation). The dynamic data comprised live stream data of performance monitoring metrics for each running task. This data consists of time-series records which include the CPU and memory usage by the container, total memory used by the container, size of data sent and received by the container over the underlying network, and the size of read/write data by the container from block devices on the host.

#### Data preparation and preprocessing

The preprocessing activities primarily focused on converting Docker’s generated monitoring statistics. First, the Docker stats were given a format flag to output the exact required container statistics. The output file was then parsed and cleaned using regex to split the column headers appropriately. The data was then converted to a Pandas DataFrame and proper datatypes were assigned to each column (e.g., timestamp column used the datetime datatype). Additionally, the units of the memory utilization columns were all standardized to Bytes.

#### Deep learning approach for training and anomaly detection

To detect anomalies, we first trained our dataset using a reconstruction AutoEncoder model to reduce the data dimension into a 30-D of embeddings. Afterwards, we input the AutoEncoder model generated output into an anomaly detection model. The following sequence of steps details our implementation: First, we split the dataset into two sets; static profile data and dynamic time-series performance monitoring information. Figure 3 shows the architecture of the encoder-decoder neural network developed for feature learning. The dynamic part of the data is fed into two-layers of a time-series RNN model encoder. This model takes batch size, number of records, and number of features as inputs and returns outputs in the form of a (1, 30) vector which is the final hidden state. The output is concatenated with the static data portion which is fed into three fully connected layers to produce the output shape of a (1, 30) vector. The decoder, on the other hand, uses the (1, 30) vector and passes it to two separate layer sequences, i.e., three fully connected layers and two RNN layers. The fully connected layers decode the static part of the input, while the RNN layers produce the dynamic time-series part. Here, a key aspect is that the encoder always provides the data input length such that the decoder knows how many time-series data points to produce.

The output of the encoder was trained over an anomaly detection model such as a one-class classification or clustering. The one-class classification algorithms are unsupervised learning algorithms that we trained using only non-anomaly data, i.e., the reduced feature set resulted from the aforementioned AutoEncoder algorithm, which can classify anomaly and non-anomaly data. These include one-class SVM, Isolation Forest, Elliptic Envelope, and Local Outlier Factor. In addition, we used different clustering algorithms, i.e., unsupervised learning algorithms trained using both anomaly and normal data. Among which we use *k*-means, Mini Batch *k*-means, Mean Shift, and Birch. All models predicted two classes or clusters, i.e., normal or anomaly. However, the performance of each model varied in terms of accuracy, precision, recall, and F1 score. We then selected the best performing model based on the calculated performance metrics for our real-time security enforcement.

### Experimental scenarios, evaluation criteria, and fault injection scheme

We conducted several experiments to evaluate our proposed security enforcement and anomaly detection framework. In these experiments, we intended to evaluate the anomaly detection scheme by investigating the performance of different anomaly detection algorithms and models. In addition, we conducted different experiments to evaluate the performance of the cloud workflow within the adopted proposed security enforcement model. We benchmarked the cloud workflow performance based on the previously proposed adaptation strategies [43]. In these experiments, we ran our designed hospital length of stay prediction workflow several times with different patient dataset sizes. The performance of the cloud workflow was continuously monitored, and adaptation strategies were executed when necessary, depending on the decision taken by the adaptation module.

#### Scenarios

We designed two scenarios for testing the proposed security enforcement model. The first scenario focused on testing the performance and accuracy of our anomaly detection and prediction model, and the second scenario evaluated the overall performance of the cloud workflow. The first scenario was implemented in two stages: First, we used the Deep Learning AutoEncoder (Section 0) to reduce dimension of the dataset containing encodings, which were then fed to the anomaly detection module. The latter implements different ML algorithms, including one-class classification and clustering algorithms. Each algorithm was evaluated and compared in terms of four different performance measures including accuracy, precision, recall, and F1 scores after applying cross fold with k-fold values of 3, 5, and 10.

In the second scenario where we evaluated the overall cloud workflow performance, we considered the CPU utilization, memory usage, network I/O bound, and disk space usage features. The cloud workflow was executed over the implemented Docker swarm environment with different resource load capacities. We compared how the adaptation module behaved in response to the detected anomalies and the performance of the cloud workflow after applying automatic adaptation strategies to respond to anomaly detection with the performance of the cloud workflow without anomaly detection application.

#### Evaluation criteria

For our AutoEncoder, we employed one of the commonly used time-series prediction models evaluation metrics, which is the Mean Square Error (MSE) defined by the following formula:

8 $$MSE=\frac{\sum_{t=1}^n{\left({y}_{pt}-{y}_t\right)}^2}{n}$$

where *y*_*pt*_ is the predicted value at time *t*, *y*_*t*_ is the actual value at time *t*, and *n* is the number of observations [44].

To further evaluate and compare our anomaly prediction models including one-class classification and clustering, we adopted different evaluation criteria including accuracy as the most intuitive measure. However, in some cases, accuracy is not always the best measure for assessing the model performance. Henceforth, we used *precision*, *recall,* and *F1 score* to compare and select the best prediction performance model. Precision is also known as the positive predictive value, which is the ratio of correctly predicted values to the total number of predicted values. Additionally, recall is referred to as the sensitivity measure and it is defined as the ratio of correctly predicted values to the number of correctly predicted values. Moreover, we have used F1 score, which is defined as the weighted average of precision and recall [45]. These common measures well represent the overall performance of our prediction models.

Furthermore, in our experimentations, we define *precision* as the ratio of the number of correctly predicted anomalies to the total number of correctly predicted anomalies and the normal incorrectly identified as anomalies. We also express *recall* as the number of anomalies correctly identified over the total number of correctly predicted anomalies and anomalies incorrectly predicted as normal. In addition, F1 score is defined as the weighted average of precision and recall. This is given by the following formulas:

9 $$Recall=\frac{true\ positives}{true\ positives+ false\ negatives}=\frac{correctly\ predicted\ anomalies}{correctly\ predicted\ anomalies+ anomalies\ incorrectly\ predicted\ as\ normal}$$

10 $$Precision=\frac{true\ positive s}{true\ positive s+ false\ positive}=\frac{correctly\ predicted\ anomalies}{correctly\ predicted\ anomalies+ normal\ data\ incorrectly\ predicted\ as\ anomalies}$$

11 $$F1\ Score=\frac{2\ast \left( Recall\ast Precision\right)}{Recall+ Precision}$$

We adopted these measurements for the obtained results to further validate our model.

#### Anomaly injection techniques

To facilitate the testing and evaluation of our anomaly detection model in consideration of various anomalies, we employed simulation-based fault injection to inject anomalous behaviors in the cloud workflow task as well as injecting false values into the monitoring log files. Existing techniques in software fault injection include runtime injections and compile-time injection [46]. Here, we adopted runtime fault injection techniques such as code insertion to simulate system stress. In this approach, we synthesized and injected different types of anomalies such as code-modification which implements fault injection during runtime and adds instructions to increase the task execution time (e.g., adding infinite loops or time delays). Faults were randomly injected in different task instances to trigger higher CPU consumption and memory usage. The objective of these anomalies was to simulate cloud workflow task attacks and cloud resources attacks. Furthermore, we simulated monitoring component attacks by injecting anomalies into the monitoring logs. These faults included heavy or light CPU utilization, memory usage, disk I/O access, and network latency which were randomly generated to synthesize log anomalies [47]. Then the behavior of the adaptation model under stress was tested to ensure the reliability and overall performance of our proposed model.

### Results and discussion

#### Deep learning AutoEncoder model evaluation

**Fig. 5:**
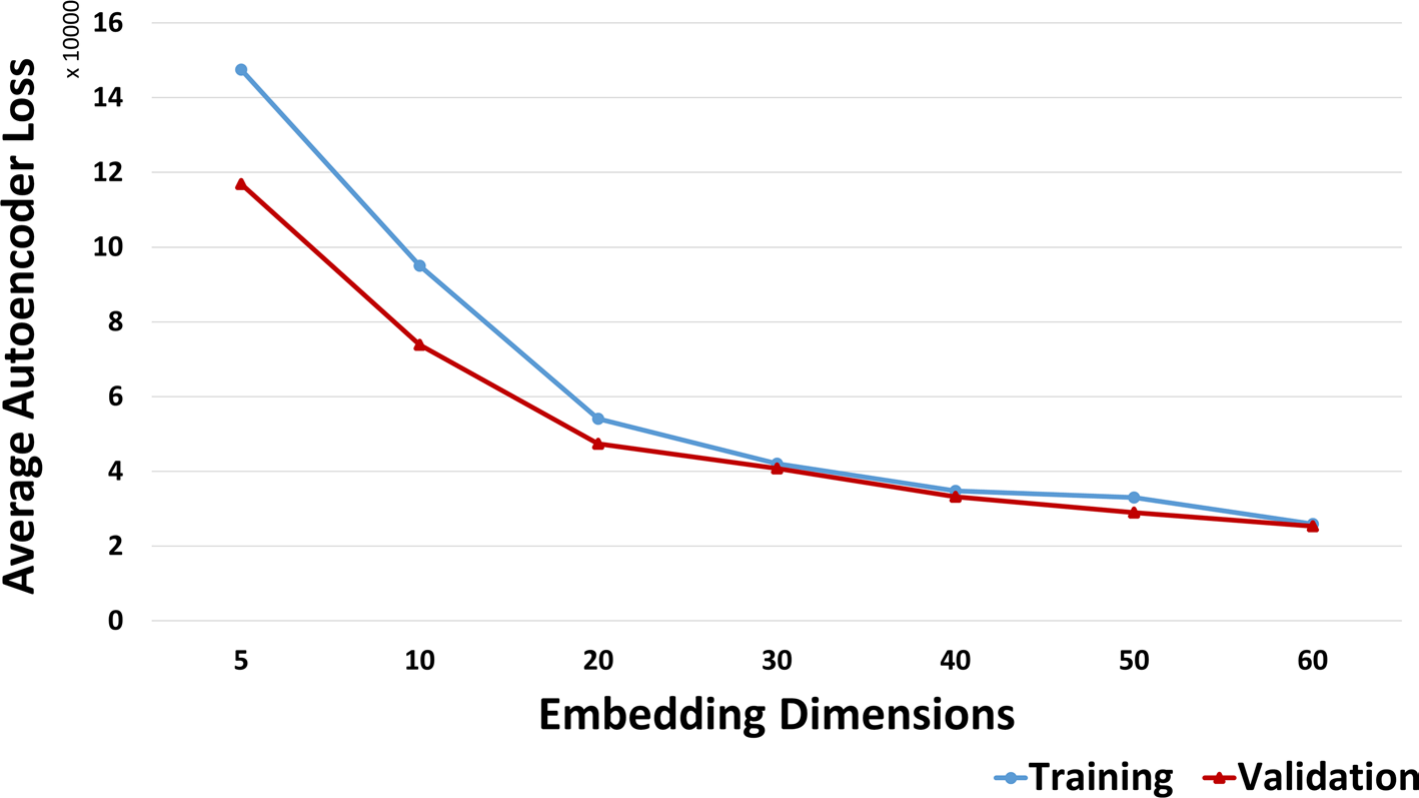
Average loss versus embedding dimensions

**Fig. 6:**
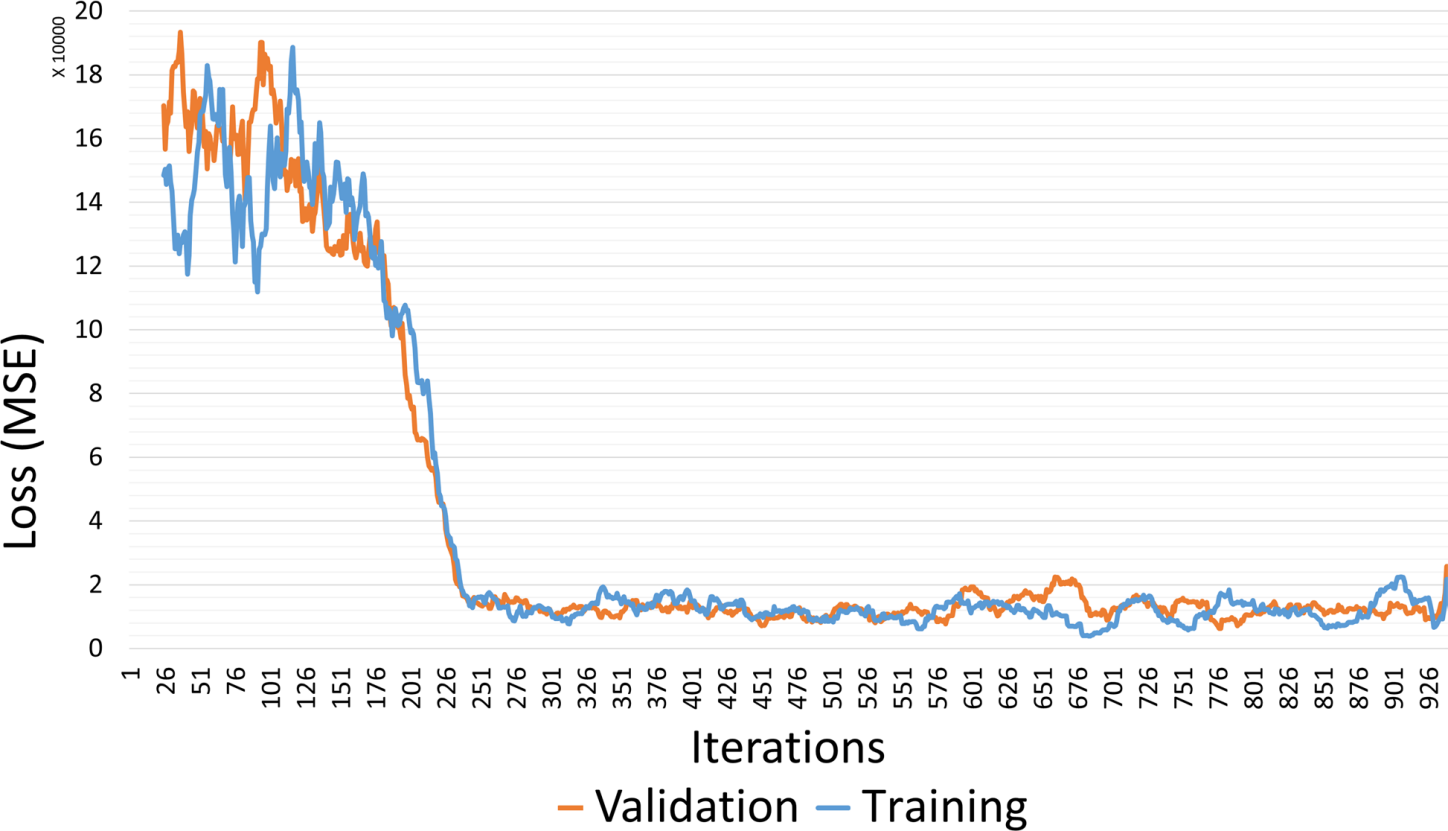
AutoEncoder reconstruction loss

In the first stage of anomaly detection, we applied deep learning with AutoEncoder to generate a reduced dimension embedding which served as an input to the anomaly detection algorithm in the second stage. We trained the AutoEncoder with normal data generated by monitoring the execution of the target case study cloud workflow. Subsequently, we selected the model that minimized the reconstruction error in the original AutoEncoder. Here, to determine embedding size, we measured the average loss while using different embedding vector dimensions during the AutoEncoder training phase. The experimental results depicted in Fig. 5 demonstrate that the average AutoEncoder reconstruction loss was reduced with higher embedding dimensions. Thus, we set the dimension of the output embedding to 30 because this provided the smallest loss value. Although higher dimension values provide slightly better loss, we set the encoder generated embedding vector size to 30 embeddings because the main objective was to reduce the dimensionality of the original dataset, which generally leads to improved accuracy. Figure 6 illustrates the AutoEncoder reconstruction loss values based on MSE while generating (1X30) vector embeddings.

#### Anomaly detection model evaluation

The main objective of this experiment was to evaluate the performance of each ML anomaly detection algorithm and select the model best suited for our dataset. We detected and predicted the anomalies in our dataset which comprised the collected cloud workflow monitoring time-series log files and the static task profile dataset. We executed the cloud workflow with normal environment settings to produce a regular dataset under the true positive conditions. Moreover, we synthesized the dataset to reflect different types of anomalies and attacks, such as task, log, or resource anomalies. For example, a task anomaly could alter a task’s behavior by increasing or reducing processing time. Whereas a log anomaly could be instantiated by injecting the monitoring logs with contradicting statistics. Furthermore, the resource anomaly included simulation of heavy load exertion on the CPU and memory resources allocated to service the cloud workflow. Here, the total number of records in both the regular and anomaly dataset was 1200 records.

We selected two main ML techniques for anomaly detection: one-class classification and clustering. For one-class classification, we compared the performance of the SVM, Isolation Forest, Elliptic Envelope, and Local Outlier Factor, each of which was subject to substantial hyperparameter tuning. For example, we ran over 800 different combinations of hyperparameter values to automatically tune the SVM model, which is discussed in the following section. All one-class classification models were trained using all regular dataset and tested with a dataset including 50% regular and 50% anomaly. On the other hand, for clustering, we evaluated *k*-means, Mini Batch *k*-means, Mean Shift, and Birch algorithms on our dataset. We trained and tested the clustering models using a dataset with a 50% regular and 50% anomaly data. Here, we adopted k-fold cross-validation to evaluate the models including classification and clustering. We applied 3-fold, 5-fold, and 10-fold cross-validation. In the following, we present our testing results.

- *One-class SVM model tuning*

**Fig. 7:**
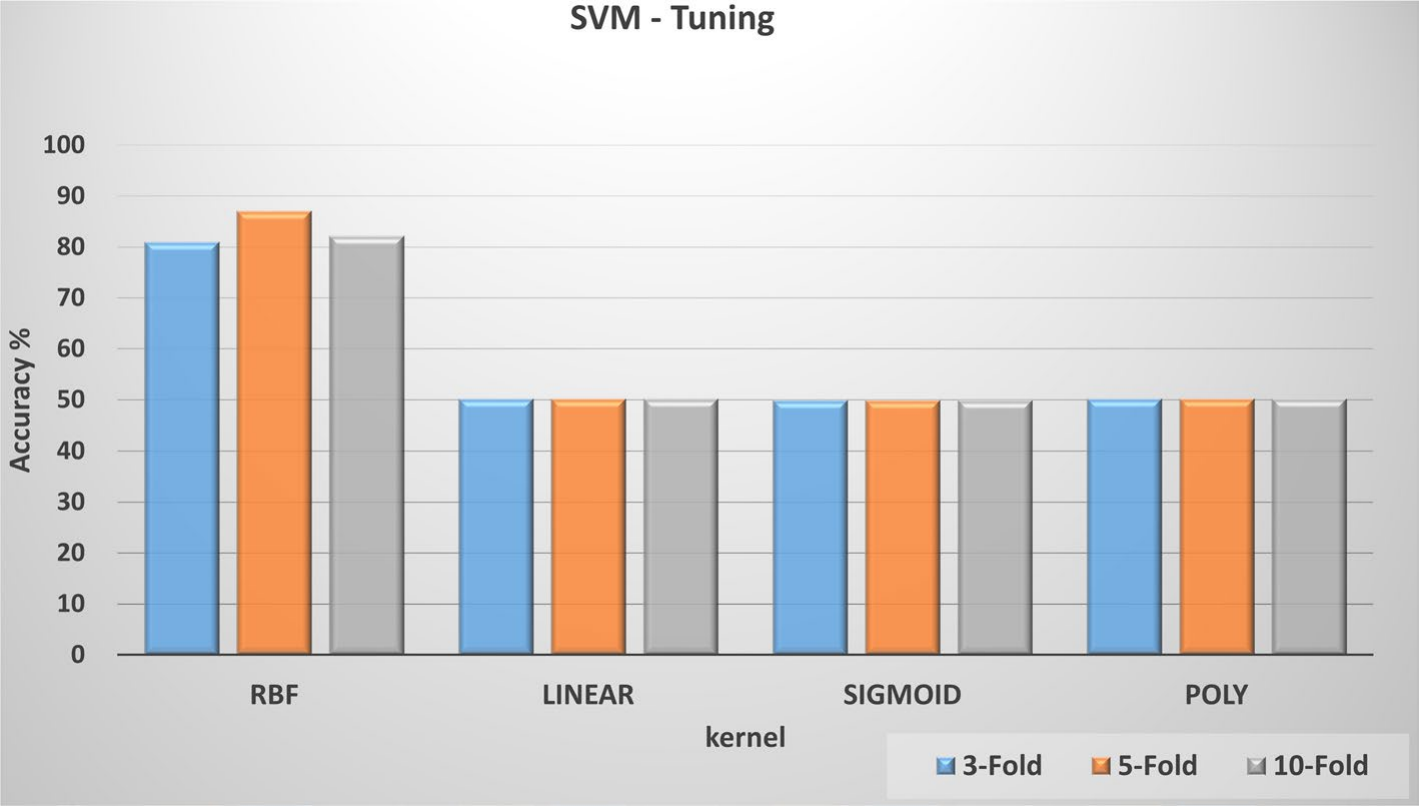
One-class SVM tuning and accuracy

In this experiment, we investigated the effect of hyperparameter tuning on the performance of the one-class SVM model. We automated the hyperparameter tuning process to quickly select the best parameter combination that gave the best accuracy. The main hyperparameters that provide the best accuracy include ***nu*** *= 0.01,* ***gamma*** *= 0.1,* ***tolerance*** *= 0.001,* ***coefficient*** *= 0****, kernel cache size*** *= 200,* and ***degree*** *(for poly) = 3*. In addition, kernel selection has the greatest effect on accuracy improvement. Figure 7 depicts the effect of different kernel parameter adoption on the accuracy using 3-fold, 5-fold, and 10-fold cross-validation. As can be seen, the RBF kernel provided the best accuracy value over sigmoid, linear, and polynomial kernels.

(b) *One-class classification models evaluation*

**Fig. 8:**
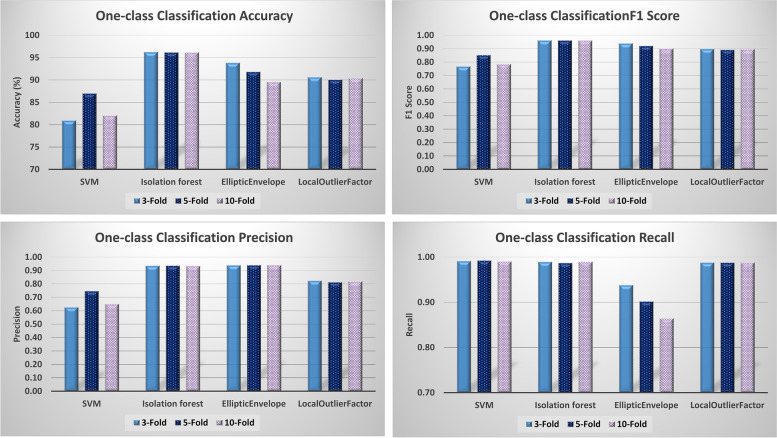
One-class classification performance evaluation

In these experiments, we compared the performance of four one-class anomaly detection classification methods including SVM, Isolation Forest, Elliptic Envelope, and Local Outlier Factor. Figure 8 depicts the performance of each algorithm in terms of accuracy, precision, recall, and F1 score. As shown, the Isolation Forest technique obtained the highest accuracy of 96.14, precision of 0.93, recall of 0.99, and F1 score of 0.96 using 10-Fold cross validation. Similar results were obtained when using 3-fold, and 5-fold cross-validation indicating that the Isolation Forest algorithm outperformed the other one-class classification algorithms.

(iii) *Clustering model evaluation*

**Fig. 9:**
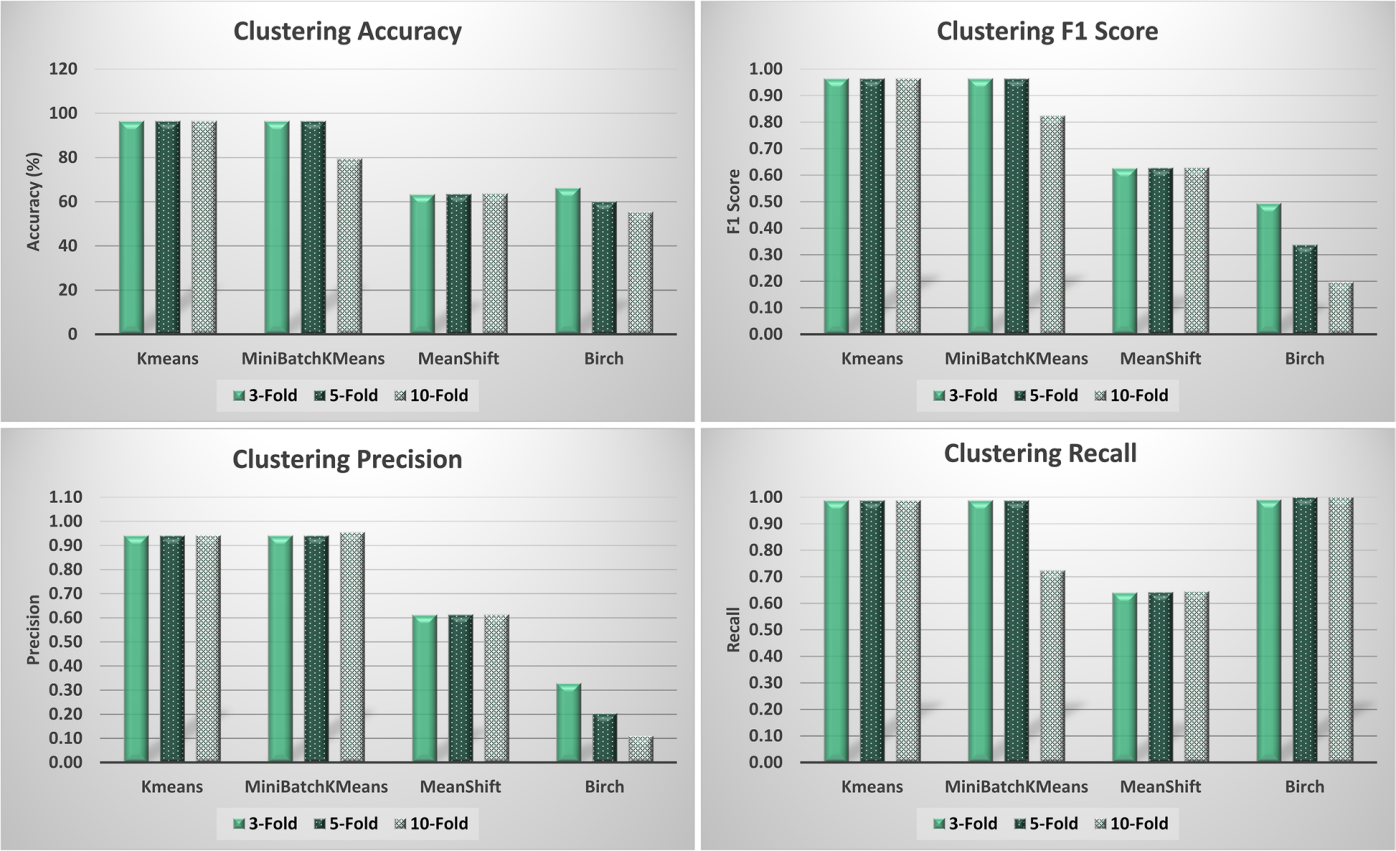
Clustering performance evaluation

Additionally, we measured the performance of different clustering algorithms namely *k*-means, Mini Batch *k*-means, Mean Shift, and Birch. Here, we trained all clustering algorithms to generate two clusters, i.e., one for regular data and another one for anomaly data. Likewise, we evaluated these algorithms with respect to accuracy, precision, recall, and F1 score while performing 3-fold, 5-fold, and 10-fold clustering as shown in Fig. 9. Generally, the *k*-means algorithm obtained the best results, demonstrating an accuracy of 96.43, precision of 0.94, Recall of 0.99, and F1 score of 0.96 using 10-fold cross-validation, and similar results were obtained with 3-fold and 5-fold cross-validation.

(iv) *Overall discussion*

We adopted two approaches for anomaly detection and prediction for security enforcement during cloud workflow execution: one-class classification and clustering. Experimental results demonstrate that clustering provided slightly better performance in terms of accuracy, precision, recall, and F1 scores over one-class classification. The *k*-means technique outperformed all other clustering algorithms. However, the isolation forest provided the best prediction performance among one-class classification algorithms and gave results that were very close to those of clustering. Considering that one-class classification training is performed using only regular data which is more likely to be the real case scenario for our cloud workflows execution rather than training with 50%:50% regular to anomaly data ratio, therefore, we recommend one-class classification specifically the Isolation Forest. Table 2 gives the anomaly detection performance results.

**Table 2:** Performance evaluation results of anomaly detection using various Machine Learning Algorithms

K-Fold	Algorithm name	Precision	Recall	Accuracy	F1 Score
**3-Fold**	SVM	0.63	0.99	80.94	0.77
	Isolation forest	0.94	0.99	96.23	0.96
	Elliptic Envelope	0.94	0.94	93.83	0.94
	Local Outlier Factor	0.82	0.99	90.63	0.90
	*k*-means	0.94	0.99	96.43	0.96
	Mini Batch *k*-Means	0.94	0.99	96.43	0.96
	Mean Shift	0.61	0.64	63.30	0.63
	Birch	0.33	0.99	66.19	0.49
**5-Fold**	SVM	0.75	0.99	86.98	0.85
	Isolation forest	0.94	0.99	96.14	0.96
	Elliptic Envelope	0.94	0.90	91.81	0.92
	Local Outlier Factor	0.81	0.99	90.06	0.89
	*k*-means	0.94	0.99	96.43	0.96
	Mini Batch *k*-Means	0.94	0.99	96.43	0.96
	Mean Shift	0.61	0.64	63.47	0.63
	Birch	0.20	1.00	60.07	0.34
**10-Fold**	SVM	0.65	0.99	82.06	0.78
	Isolation forest	0.93	0.99	96.14	0.96
	Elliptic Envelope	0.94	0.86	89.54	0.90
	Local Outlier Factor	0.82	0.99	90.38	0.90
	*k*-means	0.94	0.99	96.43	0.96
	Mini Batch *k*-Means	0.95	0.72	79.44	0.82
	Mean Shift	0.61	0.64	63.64	0.63
	Birch	0.11	1.00	55.31	0.20

#### Overall cloud workflow performance evaluation

In this section, we evaluate the overall performance of the system when using an anomaly detection approach for security enforcement over the normal adaptation strategies with no anomaly detection.

We monitored CPU utilization and memory usage of cloud workflow tasks executed over multiple nodes in the cluster. Different tasks present different utilization levels according to the nature of the task as defined by its profile. For example, a preprocessing task unitizes more CPU and memory resources than an evaluation task because preprocessing requires iterating through the entire dataset to clean and prepare the data used for training the ML model. In what follows, two experimental scenarios are discussed to demonstrate the performance evaluation of the cloud workflow.

**Fig. 10:**
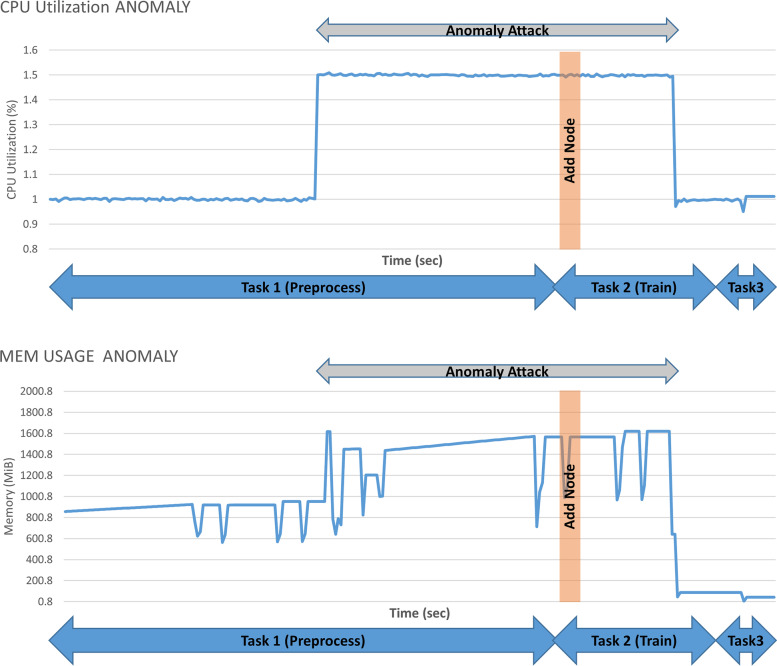
CPU utilization and memory usage during an anomaly attack

In the first scenario, we executed our cloud workflow while adopting regular quality enforcement adaptation strategies [48]. As illustrated in Fig. 10, the memory and CPU resources required to process the workflow increased over time which involved an adaptation action to add a new node after detecting that the sudden increase in resource usage was caused by an anomaly attack. In this experiment, we synthesized the log anomaly described in Section 3.2, which deceived the adaptation system, thereby resulting in unnecessary addition of resources to maintain the quality of the cloud workflow performance.

**Fig. 11:**
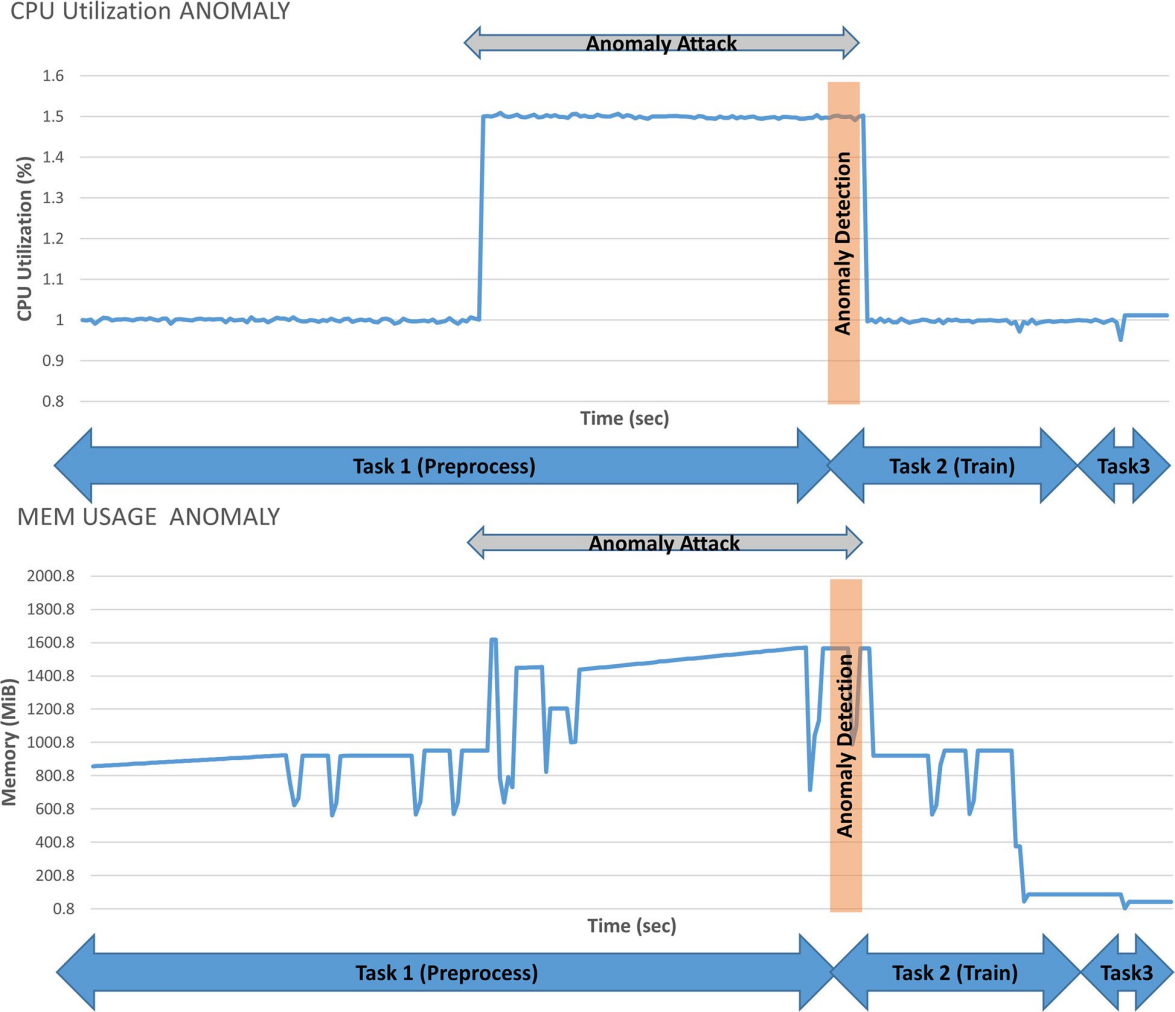
CPU utilization and memory usage with anomaly attack detection

In the second scenario, we executed the cloud workflow while embracing our new proposed security enforcement extension. Figure 11, shows that the security enforcement module detected the anomaly in task 1, thereby causing it to discard the corrupted logs and issue an action to use an older version of the logs. This action prevented the adaptation module from adding unnecessary resources.

## Conclusion

Security enforcement in cloud workflow orchestration is considered a complex research problem because of its dynamicity and changing cloud workflow execution environments. In this paper, we have proposed an architecture for cloud workflow security enforcement. The proposed architecture is applied to four main entities: the user, resources, workflow tasks, and data. A multi-modal approach incorporating deep learning, one-class classification, and clustering applied to training, anomaly detection, and prediction has also been proposed. The proposed model considers both unsupervised static and dynamic features which is a unique way of modeling features that results in better anomaly detection. It also reduces the data dimensionality which leads to better characterization of workflow tasks and thus provides a better attack prediction. Once anomalies are detected and/or predicted, adaptation measures are implemented to secure the cloud workflow execution and ensure performance. The adaptation scheme accommodates a flexible representation and planning of resource requirements over time and over the various phases of the cloud workflow execution cycle.

We conducted a set of experiments to evaluate the various features of our solutions including the application of Multi-Modal training and anomaly detection using a real COVID-19 dataset of patient health records. The proposed Multi-modal approach was formulated and tested in an experimental setup where two main scenarios were used for verification. The first scenario focused on testing the performance and accuracy of our AutoEncoder and anomaly detection model, while the second scenario was used to evaluate the overall cloud workflow performance by assessing adaptation actions taken to respond to injected anomaly detection and their impact on the performance of cloud workflow execution. Two main approaches were adopted for anomaly detection and prediction of security enforcement during the execution of the proposed workflow, i.e., LSTM-based AutoEncoder and an ML model including one-class classification and clustering. The experimental results demonstrate that clustering provides slightly better performance in terms of accuracy, precision, recall, and F1 scores over the one-class classification with *k*-means outperforming other clustering algorithms. Other experimental results of the adaptation strategy implemented to respond to detected anomalies revealed a high execution performance of the workflow. The experimental results demonstrate that the proposed architecture prevents unnecessary wastage of resources due to anomaly detection and prediction.

We plan to explore other ML algorithms to detect and predict other categories of anomalies and attacks as future work. We also plan to explore ensemble ML and natural language processing algorithms to explore new levels of cloud workflow automation, robustness, and fault tolerance.

## Data Availability

The dataset used in this study is not publicly available, however it can be provided up on request.
